# Evaluating synthetic substitutes to reduce illegal harvesting and support species recovery

**DOI:** 10.1111/cobi.70266

**Published:** 2026-03-24

**Authors:** Aditya Shekhar Malgaonkar, Gareth Whittington‐Jones, Tristan Dickerson, Maswabi Lishandu, Sarah Davies, Zoe Woodgate, Xia Stevens, Choolwe Mulenga, Gift Mulenga, Mathew Phiri, Lucky Mulenga, Manyando Mukela, Gryton Kasamu, Willem A. Nieman, Gareth Mann, Abishek Harihar, Diogo Veríssimo, Rob Pickles

**Affiliations:** ^1^ Panthera New York New York USA; ^2^ Panthera Wild Cat Conservation Zambia Limited Mumbwa Zambia; ^3^ Barotse Royal Establishment Mongu Zambia; ^4^ Wildlife Crime Prevention Lusaka Zambia; ^5^ Institute for Communities and Wildlife in Africa University of Cape Town Cape Town South Africa; ^6^ Department of National Parks and Wildlife Chilanga Zambia; ^7^ Biodiversity and Behavioral Science Team, Environmental Change Institute University of Oxford Oxford UK; ^8^ Wildlife Conservation Research Unit, Department of Biology University of Oxford Oxford UK

**Keywords:** EMMIE framework, general elimination methodology, illegal wildlife trade, Indigenous peoples, local communities, qualitative evaluation, theory‐driven evaluation, wildlife crime, comercio ilegal de vida silvestre, comunidades locales, delitos contra la vida silvestre, evaluación basada en la teoría, evaluación cualitativa, Marco EMMIC, metodología de eliminación general, pueblos indígenas, 非法野生动物贸易, 野生动物犯罪, 土著人民, 当地社区, 定性评估, 理论驱动的评估, 广义消元法, EMMIE框架

## Abstract

Providing synthetic substitutes is a widely promoted strategy to shift consumer demand away from wildlife products derived from threatened species. Yet, there is little evidence on whether product substitution prevents illegal or unsustainable harvesting and contributes to the recovery of threatened populations. Drawing on the Furs for Life Zambia initiative, which supplied synthetic furs known as heritage furs to replace leopard furs traditionally worn during Lozi royal ceremonies in western Zambia, we devised a way to test the effects and causal mechanisms of substitution. Guided by the EMMIE (effect, mechanisms, moderators, implementation, and economic cost) framework commonly used in crime prevention evaluations, we triangulated data from semistructured questionnaires, law enforcement patrols, court records, camera‐trap monitoring of leopards (*Panthera pardus*), and stakeholder interviews conducted from 2018 to 2024. We used qualitative analyses and the general elimination method to assess plausible alternative explanations for leopard recovery. By 2024, adoption of synthetic furs among leopard fur users exceeded 80%, and self‐reported ownership of authentic leopard furs declined by 78%. Patrol detections of leopard poaching incidents decreased, and camera‐trap density estimates increased from an average of 2.7 to 3.8 leopards per 100 km^2^ across the focal landscape. An integrated mechanism of change, derived from stakeholder perspectives, indicated that reduced demand from substitution was reinforced by concurrent counter‐poaching and counter‐trafficking operations. Our results provide the first empirical link between a demand‐reduction initiative based on synthetic substitutes and measurable population recovery of species.

## Abstract (es)

Evaluación de sustitutos sintéticos para reducir la recolección ilegal y apoyar la recuperación de especies

**Resumen:**

El abastecimiento de sustitutos sintéticos es una estrategia ampliamente promovida para desviar la demanda de los consumidores de productos derivados de especies amenazadas. Sin embargo, hay escasas pruebas de que la sustitución de productos impida la captura ilegal o insostenible y contribuya a la recuperación de las poblaciones amenazadas. Basándonos en la iniciativa Furs for Life Zambia, que suministró pieles sintéticas conocidas como “pieles tradicionales” para sustituir las pieles de leopardo que se usaban tradicionalmente en las ceremonias reales de Lozi, en el oeste de Zambia, ideamos una forma de comprobar los efectos y los mecanismos causales de la sustitución. Guiados por el marco EMMIC (efecto, mecanismos, moderadores, implementación y coste económico), comúnmente utilizado en las evaluaciones de prevención del delito, triangulamos los datos de cuestionarios semiestructurados, patrullas policiales, registros judiciales, monitoreo con cámaras trampa de leopardos (Panthera pardus) y entrevistas a las partes interesadas realizadas entre 2018 y 2024. Utilizamos análisis cualitativos y el método de eliminación general para evaluar explicaciones alternativas plausibles para la recuperación del leopardo. En 2024, la adopción de pieles sintéticas entre los usuarios de pieles de leopardo superó el 80 %, y la propiedad autodeclarada de pieles de leopardo auténticas disminuyó en un 78 %. Las detecciones de incidentes de caza furtiva de leopardos por parte de los patrullajes disminuyeron, y las estimaciones de densidad de cámaras trampa aumentaron de una media de 2,7 a 3,8 leopardos por cada 100 km2 en todo el paisaje focal. Un mecanismo integrado de cambio, derivado de las perspectivas de las partes interesadas, indicó que la reducción de la demanda por la sustitución se vio reforzada por operaciones simultáneas contra la caza furtiva y el tráfico ilegal. Nuestros resultados proporcionan el primer vínculo empírico entre una iniciativa de reducción de la demanda basada en sustitutos sintéticos y la recuperación cuantificable de la población de especies.

## 评估利用合成替代品减少非法捕猎并支持物种恢复的成效

**【摘要】** 提供野生动物制品的合成替代品是一种得到广泛推广的策略, 旨在引导消费者转移对来自受威胁物种的野生动物制品的需求。然而, 关于替代产品能否有效遏制非法或不可持续捕猎, 并促进受威胁物种种群恢复的证据尚显不足。本研究以赞比亚“生命皮草(Furs for Life)”计划为案例, 设计了验证替代效应及因果机制的评估方案。该计划通过提供名为“传统皮草”的合成皮草, 来替代赞比亚西部洛齐族皇家仪式中传统使用的豹皮。我们采用犯罪预防评估中常用的EMMIE框架(效果、机制、调节因素、实施情况及经济成本), 对2018至2024年间获得的半结构化问卷、执法巡逻记录、法庭档案、豹子(*Panthera pardus*)红外相机监测数据, 以及利益相关者访谈, 进行了数据三角验证。研究还通过定性分析与广义消元法, 评估了豹子种群恢复的其他可能解释。豹皮使用者中采用合成皮草的比例超过80%, 自我报告持有真豹皮的比例下降78%。巡逻队发现的豹子盗猎事件减少, 而研究区域内红外相机数据估计的豹子密度从每100平方公里2.7只增至3.8只。基于利益相关者视角构建的综合变化机制表明:替代品出现导致的需求下降, 与同步开展的反盗猎及反走私行动形成协同效应。本研究首次实证揭示了基于合成替代品的需求削减措施与可检测的物种种群恢复之间的关联。**【翻译:胡怡思;审校:聂永刚】**

## INTRODUCTION

Illegal and unsustainable wildlife trade is a leading proximate threat to global biodiversity, accelerating population declines and local extinctions (Hinsley et al., 2023; Morton et al., 2021). A meta‐analysis of 506 sites and 145 vertebrate species found that exploited populations were, on average, 60% smaller than matched controls and 16% were locally extirpated (Morton et al., 2021). Conventional, enforcement‐centered responses such as trade bans, protected area designations, and law‐enforcement patrols have slowed losses but on their own often fail to halt declines (Geldmann et al., 2019; Hiller & ’t Sas‐Rolfes, 2025). Many biodiversity hotspots, particularly in South America, sub‐Saharan Africa, and Southeast Asia, are located in lower‐income nations where wildlife agencies remain chronically understaffed and underresourced (Appleton et al., 2022). Under such conditions, prohibitions can inflate black market prices, attract organized crime, and displace hunting (Hiller & ’t Sas‐Rolfes, 2025). Coercive approaches can also alienate Indigenous peoples and local communities (IPLCs) who depend on wildlife for nutrition, income, or cultural identity, thereby undermining legitimacy and effectiveness (Torrents‐Ticó et al., 2023).

These limitations have prompted growing interest in substitution strategies that provide synthetic or alternative products that meet user needs without wild harvesting (Broad & Burgess, 2016). If substitutes are affordable, accessible, and culturally acceptable, they can reduce the scarcity premium that fuels poaching (Thomas‐Walters et al., 2021) while preserving symbolic or ritual functions that facilitate behavioral change (Rock & MacMillan, 2022). Recent examples demonstrate the growing potential of wildlife product substitution across ceremonial, medicinal, and culinary markets in Asia (Choy et al., 2024; Davis et al., 2022; Dhar, 2023) and in South Africa, where the Furs for Life initiative halved leopard fur use within a decade (Naude et al., 2020). Despite these promising cases, rigorous evaluations remain scarce, creating uncertainty about the effectiveness, durability, and potential unintended effects of substitution interventions.

This evidence gap reflects a broader shortfall in counter‐wildlife‐crime research. A systematic review of 123 field‐based interventions found only five with evaluation designs capable of attributing causation, none of which tested product substitution strategies (Delpech et al., 2021). Gaps are most acute where interventions intersect with IPLC ceremonial practices that impose strong authenticity requirements (Torrents‐Ticó et al., 2023). To date, most peer‐reviewed substitution studies focus on consumer attitudes, stated intentions, or adoption rates (Choy et al., 2024; Davis et al., 2022; Naude et al., 2020), whereas rigorous evaluations of conservation outcomes such as reduced harvesting or population recovery remain rare (Rytwinski et al., 2021).

Evaluators, therefore, must ask whether interventions curb consumption, measurably diminish direct threats to targeted species populations, and promote stable or increasing population trends, while also illuminating mechanisms linking actions to outcomes. This is challenging because as illegal trade is covert, baseline data are sparse, and multiple interventions often overlap (Baylis et al., 2016). Quantitative indicators such as snare detections, seizure volumes, and density estimates show what changed, but seldom why (Johnson et al., 2015). Changes in seizures and arrests may reflect variations in detection effort rather than actual shifts in the illegal trade itself (Paudel et al., 2022). Evaluation designs integrating qualitative insights from interviews, focus groups, and document analysis with quantitative data are increasingly advocated to connect observed changes to underlying mechanisms (Zavaleta Cheek et al., 2023).

The EMMIE framework (effect, mechanism, moderators, implementation, and economic cost) provides a structured template for such evaluations (Johnson et al., 2015). For example, a recent assessment of counter‐poaching patrols in Malaysia linked specific patrol tactics (mechanism) to reductions in snaring (effect) and identified landscape and offender characteristics that moderated success (Lam et al., 2023). The general elimination method (GEM) complements EMMIE by systematically ruling out rival explanations until the most plausible causal narrative remains (Salazar et al., 2019; Scriven, 2008). Embedding GEM within the effect–mechanism–moderator steps provides a transparent and systematic chain of reasoning that strengthens causal inference. This approach is particularly valuable in wildlife trade contexts, where randomized or perfectly matched controls are rarely feasible and where multiple external factors can obscure attribution (Baylis et al., 2016).

The ceremonial use of leopard (*Panthera pardus*) furs in western Zambia illustrates the opportunity and challenges for demand reduction through synthetic substitutes. Each year, the Barotse Royal Establishment and the Lozi people mark the flood cycle of the Barotse Floodplains with the Kuomboka and Kufuluhela ceremonies. During the main Kuomboka, around 200 royal paddlers ferry the Litunga, the King of the Lozis, on a black‐and‐white Nalikwanda barge while wearing red berets trimmed with lion mane and lipatelo skirts made of leopard, serval (*Leptailurus serval*), genet (*Genetta* spp.), civet (*Civettictis civetta*), and, rarely, cheetah (*Acinonyx jubatus*) furs. Leopard furs symbolizing power, courage, and grace are preferred, whereas other spotted carnivore furs are alternatively used in the absence of leopard fur or added to enhance appearance. An estimated 1000 prospective paddlers vie for paddler places each year, generating demand for an estimated 150–250 wildlife fur garments annually (Panthera, 2022a).

Paddlers obtain leopard furs by hunting directly or trading with local poachers. All such activity is illegal without a permit under the Zambian Wildlife Act (2015). Interviews with key community contacts, questionnaires with paddlers, and wildlife seizure records confirm that the nearby Greater Kafue ecosystem (GKE) is the principal source of ceremonial leopard skins (Panthera, 2022a). Covering ∼66,000 km^2^ of miombo woodlands, grasslands, and wetlands, the GKE includes the Kafue National Park and surrounding game management areas. The Department of National Parks and Wildlife (DNPW) partners with Panthera, African Parks, Game Rangers International, and Musekese Conservation to conduct counter‐poaching patrols and counter‐trafficking interventions. Although offenders caught hunting or transporting leopard furs may be arrested, law‐enforcement agencies refrain from seizing garments at the Lozi ceremonies out of cultural sensitivity. The poaching and trafficking of leopard fur are believed to be closely linked to ceremonial demand for leopard fur (Whittington‐Jones et al., 2020).

To stem this pressure, the late Senior Chief Inyambo Yeta invited Panthera in 2017 to replicate South Africa's Furs for Life model in Western Zambia. The resulting project, initially named Saving Spots, now Furs for Life Zambia, began distributing high‐fidelity synthetic leopard furs in 2019. Implementation (2019–2024) coincided with intensified patrols and counter‐trafficking operations and overlapped with COVID‐19 lockdowns, creating a complex setting in which multiple interventions, deep‐rooted cultural norms, and external shocks interact.

Our study is the first rigorous evaluation of a wildlife product substitution strategy that tracks outcomes along the full supply chain—from consumer demand, trafficking, and poaching to population trends of the target species. By analyzing multiple data sources and explicitly accounting for contextual complexity, we sought to determine whether synthetic substitutes can curb illegal trade and drive measurable species recovery.

## METHODS

### Evaluation approach

**FIGURE 1:**
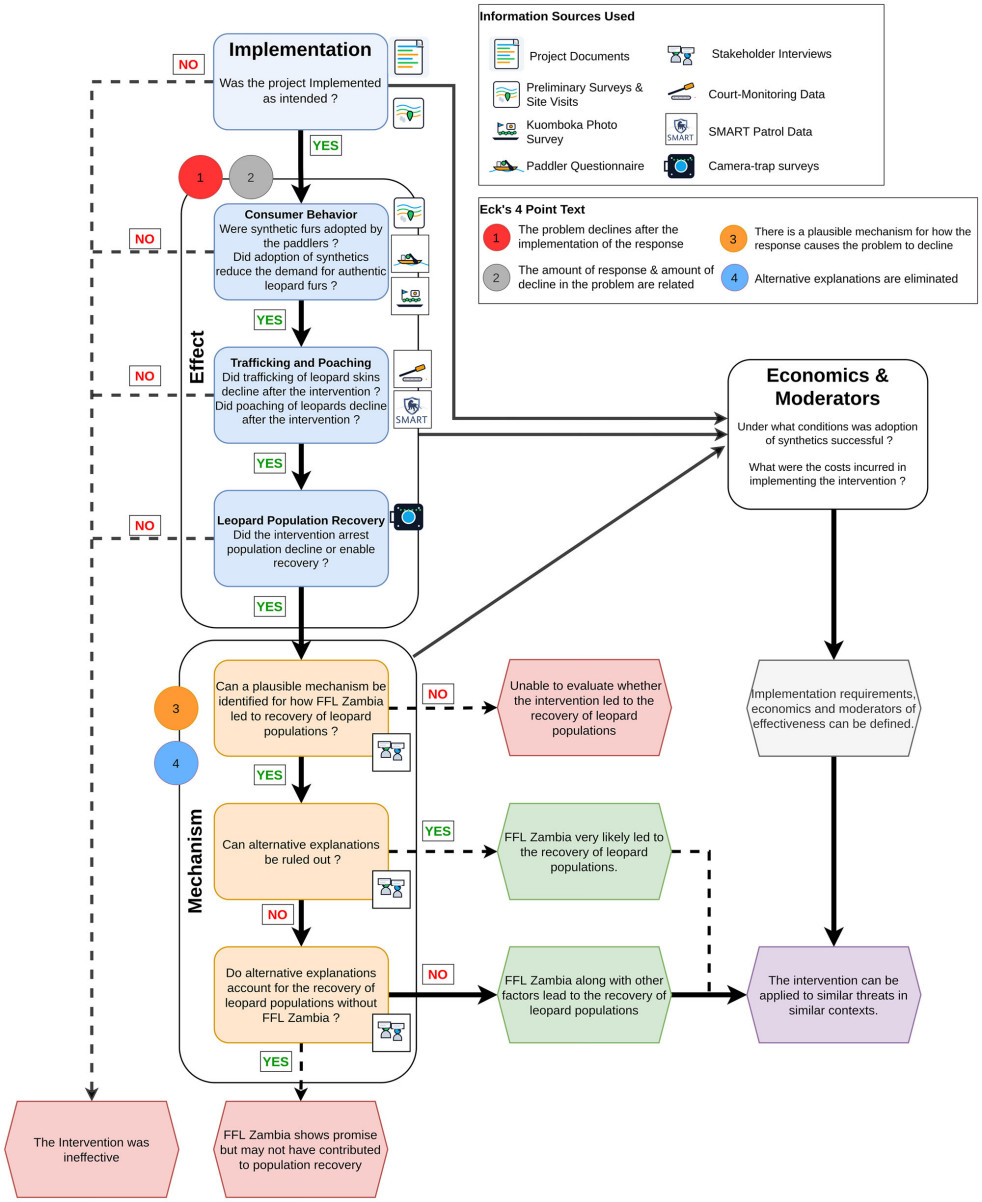
Workflow documenting the EMMIE (effect, mechanisms, moderators, implementation, and economic cost) evaluation approach and information sources used in this study of synthetic leopard fur substitution intervention (FFL, Furs for Life).

We used the EMMIE framework to structure our evaluation, integrate multiple evidence streams, and link intervention delivery to ecological outcomes. To strengthen causal inference, we nested two complementary methods within this framework: Eck's (2017) criteria for determining causality and the GEM to investigate alternative explanations and develop an integrated mechanism for change (Figure 1).

First, we verified that the synthetic‐fur rollout was implemented as planned, documenting challenges and adaptations; this established the factual basis for analyzing the effect and mechanism. Next, we quantified intervention effects at three nested levels: consumer demand for leopard furs, trafficking or poaching pressure, and leopard population trend, thereby tracing the complete causal chain predicted by the project logic. At this stage, Eck's (2017) criteria allowed us to judge whether change followed implementation and whether the magnitude of change matched the scale of the intervention. We then applied GEM to determine whether a credible mechanism linked the intervention to the observed changes and whether alternative explanations could account for the observed outcomes, thereby fulfilling all four of Eck's (2017) causality criteria.

Finally, we identified moderators (i.e., context‐specific factors that facilitated synthetic fur adoption and downstream leopard recovery) and addressed the economics dimension by estimating total project costs and expressing cost‐effectiveness as the cost per leopard use prevented.

We assessed whether Furs for Life Zambia was implemented as designed by tracking project implementation from the issuance of the royal declaration and synthetic fur distribution through to their use at Kuomboka ceremonies. The implementation rollout was reconstructed from project reports, and the impact of delays caused by disruptions such as the COVID‐19 pandemic and adaptation measures used were documented.

### Consumer behavior change and change in leopard fur use

A preliminary survey was conducted in 2018 to establish a baseline, during which two researchers recorded the number of leopard, serval, and other carnivore furs worn by paddlers at the Kuomboka ceremony. Following project initiation, four structured questionnaire surveys targeting Lozi royal paddlers or prospective paddlers (2020: *n* = 86; 2021: *n* = 23; 2022: *n* = 57; 2024: *n* = 110) were used to document their awareness of synthetic furs, perceptions of synthetic furs, intention to acquire authentic leopard furs, current fur ownership, and use of authentic leopard furs at the Kuomboka ceremony. The questionnaires were administered in the Lozi language by a local Lozi researcher (M.L.) familiar with cultural context and practices (questions in Appendix S1).

For each year, we used exact binomial tests to examine whether the observed proportions for each of the five binary response variables differed significantly from the random expectation of 0.50 and reported the associated 95% confidence intervals (CIs) and *p* values (Sokal & Rohlf, 2012). To assess temporal changes in the same binary response variables, we fitted generalized linear models (GLMs) with a binomial error distribution and a logit link function, treating year as a continuous predictor. Regression coefficients (β) and their associated *p* values were used to evaluate temporal trends in awareness, perception, intention, ownership, and use (Crawley, 2013). Model assumptions were assessed using residual diagnostics and visual checks for overdispersion. Statistical significance for all tests was accepted at α = 0.05.

To assess the effect of demand reduction on authentic fur ownership, we estimated fur ownership under a zero‐procurement scenario, accounting for natural decay but no new acquisitions. The expected decline was estimated from the mean replacement period reported by paddlers, assuming a normal distribution of fur ages with the mean fur age set to half the replacement period (μ = *x*⁄2) and the standard deviation scaled such that nearly all furs fell within the expected 0‐ to *x*‐year lifespan (σ = *x*⁄6). Observed changes in reported ownership were then compared with the estimated expected decline. All analysis was conducted using R statistical software 4.4.1 (R Core Team, 2024).

The interviews and questionnaire surveys were approved by the Research Ethics Committee, Faculty of Science, University of Cape Town, in 2019 (reference number FSREC 92–2019). Law enforcement support activities, implementation of SMART software, and monitoring of the leopard population in the GKE were conducted jointly under the terms of a memorandum of understanding signed by Panthera Wild Cat Conservation Limited, Zambia, and the DNPW, Zambia, in December 2019. No personally identifiable information of human participants or local communities is disclosed, and all responses regarding natural resource use or potentially illicit activities were anonymized to protect the confidentiality and safety of respondents.

### Trafficking and poaching trends

We used information from Wildlife Crime Prevention Zambia's court‐monitoring program covering wildlife‐crime‐related cases across 16 courts in western and southern Zambia (1069 case records, 2018–2024) and law enforcement patrol data collected from the GKE (196,600 km foot patrols, 2018–2022) to index trafficking and poaching, respectively. Poaching detections from GKE were examined descriptively due to limited data.

### Leopard density trends

Twenty‐four camera‐trapping surveys were conducted in five sectors across the GKE from 2018 to 2024. To investigate temporal changes in leopard density (individuals/100 km^2^), we applied maximum likelihood multisession spatial capture–recapture models (Borchers & Efford, 2008), treating each year as a separate session. Models estimated population density (*D*) by incorporating the spatial locations of individual detections to estimate baseline detection probability (*g*0) and the spatial decay parameter (σ) (Gerber & Parmenter, 2015). Although spatial capture–recapture models are typically used to estimate density at a single point in time, multisession (i.e., multiyear) data can be modeled to describe population trends (Royle et al., 2013). In multisession spatial capture–recapture models, parameters such as *g*0 and σ are shared across sessions, thereby smoothing excess sampling variance and increasing model accuracy (Rogan et al., 2019). To account for sex‐specific capture heterogeneity (Sollmann et al., 2011), all models included sex as a covariate on σ and were modeled using a partially observed finite mixture. All spatial capture–recapture models were fitted using the package secr 5.2.4 (Efford, 2025). Because each session represented a closed population, we specified the total area of leopard habitat sampled per session, by creating a buffer around each station equal to four times σ. We restricted this model state space to avoid modeling density within urban areas or water bodies (Plummer et al., 2017).

We estimated the finite growth rate, or population trend, as the change in leopard density between sessions (λ*_t_*), by reparametrizing the density model such that

$$\lambda_{t}=\frac{D_{t+1}}{D_{t}},$$

where *D_t_* is density at year *t* (Efford, 2025; Hadi et al., 2025).

### Mechanism and alternative explanations

To identify mechanisms through which demand reduction may have contributed to leopard recovery in the GKE, we conducted 22 semistructured interviews with purposively selected stakeholders from three groups central to the poaching issue: Lozi community leaders and paddlers, DNPW officers, and conservation nongovernmental organization (NGO) staff (Appendix S2). Interviews were conducted in person or via video conferencing from February to April 2024 in English and in Lozi with an interpreter for community members.

After informed verbal consent, each session began with background questions, followed by a leopard population trend exercise. Respondents were shown a graph of leopard density estimates for 2018–2022 and a deck of 12 cards representing potential causal factors drawn from global leopard assessments (Jacobson et al., 2016; Stein et al., 2023), regional case studies (Panthera, 2022a, 2022b), and the field experience of authors (G.W.J., T.D.). Interviewees sorted the cards into factors believed to have positively influenced leopard numbers (Category A), factors not contributing positively (Category B), and unsure or unknown (category C) and could add new factors (Appendix S2). They then ranked Category A factors from most to least influential while explaining their reasoning. Probing questions explored causal pathways and interactions. Card order was shuffled between interviews. The exercise was replicated with digital cards in a shared Miro workspace for online sessions. Rankings and narratives were recorded in a field notebook; factor rankings and respondent metadata were compiled in a spreadsheet, and narratives were transcribed into a text document.

Narrative data were manually coded to standardize recurring phrases and iteratively identify themes. We combined responses to generate frequency scores for each factor, stratified by stakeholder group, and evaluated supporting rationale. Following the GEM (Salazar et al., 2019; Scriven, 2008), we constructed stakeholder‐specific mechanisms of change, retaining factors cited by at least one third of respondents in each group. These were merged to form an integrated mechanism, retaining factors endorsed by at least two of the three groups and refining causal linkages. The mechanism was cross‐validated against analysis of paddler questionnaires, patrol records, wildlife crime court cases, and project case studies (Panthera, 2022a, 2022b). Expected outputs derived from the mechanism were compared with observed trends to assess the plausibility of causal links among synthetic fur adoption, complementary interventions, and leopard recovery.

### Moderators of synthetic fur adoption

Questionnaire responses of paddlers on their opinion on synthetic furs were stratified by their responses on their desire to acquire authentic leopard furs, reported ownership of authentic leopard furs, hunting for leopard fur, and use of leopard furs at Kuomboka. Respondent covariates (age, experience, employment, and education status) were examined to identify contextual moderators of synthetic fur adoption.

### Economics of implementation

We estimated the cost per adult leopard use prevented by dividing total project expenditure by the number of whole leopard furs no longer required for ceremonial garments due to the intervention. The leopard‐use‐prevented estimate may not directly equate to leopard deaths averted, as leopard furs used by the paddlers may originate from opportunistic scavenging of leopard bycatch in bushmeat snares. However, this provides a practical benchmark for assessing the cost‐effectiveness of synthetic fur substitution.

Project expenditure had three components: audited start‐up costs during the initiation phase (2018–2020); actual maintenance outlays over the first 5 fiscal years (2020–2024); and the net present value of projected maintenance costs for the subsequent 5 years (2025–2029), discounted at 8% annually to account for high local inflation.

Leopard furs prevented from ceremonial use were estimated using a Fermi approach that decomposed the calculation into a set of observable parameters: the annual number of prospective paddlers, the preintervention proportion wearing authentic furs, the lifespan of authentic garments, the number of garments per leopard, the 10‐year lifespan of synthetics, and the annual adoption rate of synthetic furs. These inputs were combined to estimate annual demand for leopard furs and the number prevented each year through substitution. Summing these across the 2020–2029 evaluation window yielded the potential total number of adult leopards prevented from ceremonial use. For each parameter, mean or median, minimum, and maximum values were applied to generate likely, conservative, and optimistic scenarios, producing corresponding cost‐per‐leopard estimates (Appendix S3).

## RESULTS

### Implementation process

Consultations with the Barotse Royal Establishment guided the design of realistic and culturally appropriate synthetic garments known as heritage furs, modeled on authentic leopard patterns. The first batch was unveiled at the public launch of Furs for Life Zambia in August 2019 and worn by 70 royal paddlers accompanying the Litunga during the Kupuwana ceremony in September 2019. Following user feedback, batches were refined, and by early 2022, a total of 1350 heritage furs had been delivered to the Barotse Royal Establishment.

In December 2019, the Litunga issued a royal decree mandating that only synthetic furs be worn at all official Lozi ceremonies as a means to uphold cultural tradition while safeguarding wildlife. The decree was broadcast on the radio by the Barotse Royal Establishment's Prime Minister and reiterated at a November 2019 ceremony, providing institutional legitimacy for substitution.

The COVID‐19 pandemic disrupted ceremonies in 2020 and 2021, delayed fabric shipments, and constrained in‐person outreach, posing risks to sustained community engagement. The project adapted by shifting to online communication through WhatsApp messaging and YouTube videos to promote the use of synthetic furs through positive, tradition‐centered messaging. Synthetic furs were provided to paddlers for the 2022 Kuomboka Ceremony and the 2024 event following the cancellation of Kuomboka in 2023. A local outreach officer maintained year‐round engagement with communities, conducted surveys, and distributed educational materials, whereas a project coordinator oversaw logistics and liaised with the Barotse Royal Establishment and Panthera. Throughout implementation, senior traditional leaders played a central role in sustaining cultural acceptance and legitimacy.

The Barotse Royal Establishment retains custody of the synthetic furs and oversees their distribution to paddlers before each ceremony, as well as their retrieval and reuse afterward. Given an estimated lifespan of 8–10 years per garment, replacement is not anticipated until approximately 2027–2029. In the interim, this project aims to support a transition toward a self‐sustaining model by involving local communities in tailoring and commercial sale of synthetic furs.

### Consumer behavior change and change in leopard fur use

**FIGURE 2:**
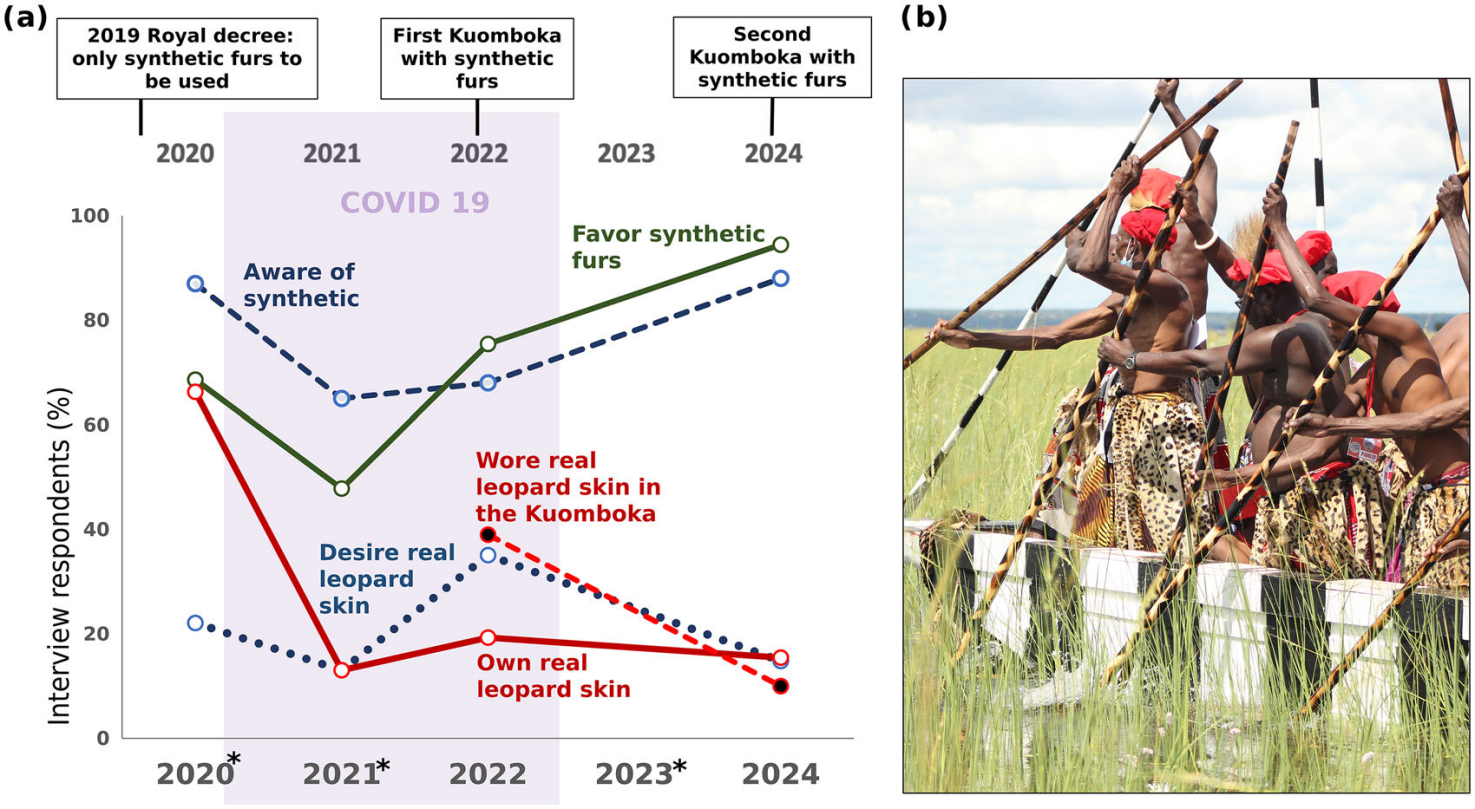
(a) Change in perceptions among prospective paddlers (i.e., Lozis wanting to paddle the royal barge) interviewed about key events in the Furs for Life Zambia intervention to prevent killing of leopards and (b) royal paddlers wearing synthetic furs during the 2022 Kuomboka (i.e., annual Lozi royal ceremony marking the Litunga's symbolic journey from the flooded Barotse Floodplain to higher ground) (*, years with no Kuomboka).

Across the four surveys (2020–2024), awareness of synthetic furs was consistently high; 82% of prospective paddlers (226 of 279) were aware of them. This percentage was significantly greater than random expectation (*p* < 0.01, 95% CI 78–100). Although awareness dipped in 2021, it increased significantly from 2021 to 2024 (β_year_ = 0.51, *p* < 0.01) (Figure 2). Attitudes mirrored this pattern with 79% of respondents (215 of 276) expressing a favorable opinion of synthetic furs (exact binomial, *p* < 0.01, 95% CI 73–100). Following a transient decline in 2021, approval climbed to 95% in 2024 (β_year_ = 0.95, *p* < 0.01).

The desire among paddlers surveyed to acquire authentic leopard furs remained low and stable. Only 21% of paddlers wished to obtain authentic fur, significantly below the 50% benchmark (*p* < 0.01, 95% CI 17–26), and this percentage showed no temporal trend (β_year_ = −0.10, *p* = 0.25). Actual ownership, however, declined sharply (Figure 2). The use of authentic furs during the Kuomboka ceremony plummeted (β_year_ = −1.69, *p* < 0.01); 39% of royal paddlers wore authentic leopard furs in 2022, whereas 10% wore authentic leopard in 2024 (a 74% decline over 2 years) (Figure 2).

In 2020, 66% of paddlers surveyed (57 of 86) owned at least one leopard fur. By 2022, self‐declared ownership had declined to 19%, a 71% reduction over 2 years (β_year_ = −0.72, *p* < 0.01), and by 2024 to 15%, representing a 78% reduction over 4 years (β_year_ = −0.59, *p* < 0.01). The mean replacement period for authentic furs reported by paddlers was 4.25 years. Under a zero‐procurement scenario, the expected decline was 43% after 2 years (2020–2022) and 99% after 4 years (2020–2024). The observed decline during the initial 2 years (71% in 2022) exceeded the expected decline (43%), whereas by 2024, the overall observed decline (78%) was less than the 99% expected in the absence of new acquisitions.

### Trafficking and poaching trends

The court monitoring recorded 1069 wildlife‐related arrest and seizure incidents from 2018 to 2024, including 48 leopard‐related cases involving the poaching of at least 56 individual leopards. The number of leopard‐related seizures declined significantly from 11.25 per 100 wildlife crime cases in 2018 to 2.88 per 100 cases in 2024 (β_year_ = −0.30, *p* = 0.02). However, the data do not suggest that seizures of other spotted carnivores either declined or increased during the same period (β_year_ = 0.12, *p* = 0.432).

**FIGURE 3:**
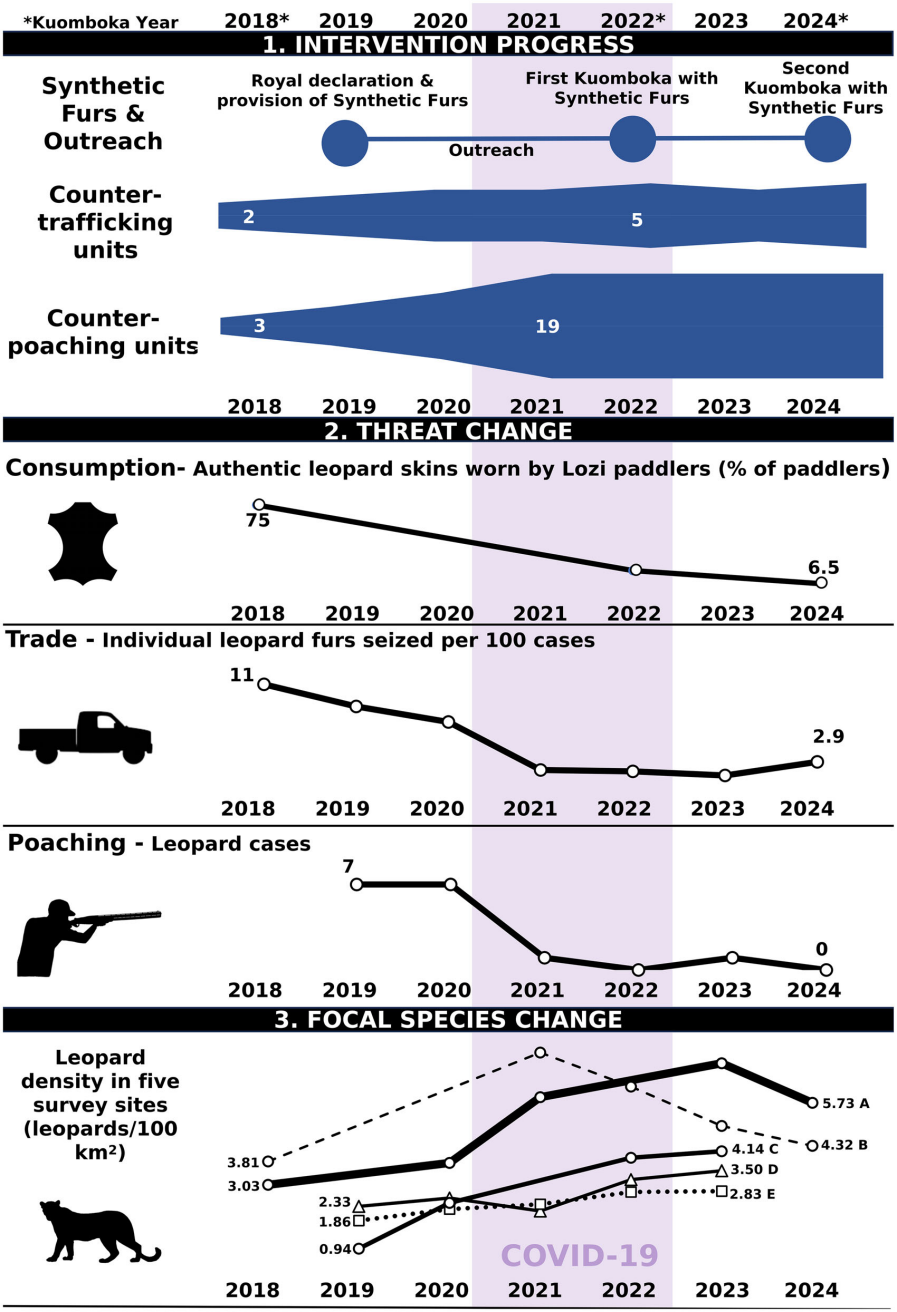
Evolution of interventions to counter‐poaching and illegal wildlife trade, change in leopard trafficking and poaching incidents, and leopard density trends at five sites in Greater Kafue ecosystem, where the leopard population was monitored (A, Kafue central; B, Mumbwa game management area [GMA]; C, Mulobezi GMA; D, Kafue north, E, Kafue south; *, years with no Kuomboka ceremony).

Seven leopard poaching incidents were reported from GKE in 2019, four of which were recorded by patrols, and three incidents were recorded through court monitoring data (Figure 3). In 2020, the number of reported poaching incidents remained unchanged, with six incidents recorded through court monitoring and one through ranger patrols. However, from 2021 to 2024, patrols recorded just two incidents of leopard poaching, indicating a marked decline.

### Leopard density trends

Estimates from multisession spatial SCR models (Figure 3), as well as λ_overall_ (i.e., mean λ_t_ for each sector) (Table 2), indicated leopard population growth across the GKE (with λ_overall_ >1.0 for most sectors), with little to no fluctuations in annual density estimates. Although λ_overall_ was greater than 1 for both GMAs (Table 2), there was an apparent downturn in population growth in leopard density for Mumbwa GMA (after a peak in 2021) (Figure 3). Mumbwa's 2024 density estimate did not significantly differ from the baseline density (4.32/100 km^2^ [95% CI 3.24–5.76] vs. 3.81/100 km^2^ [95% CI 2.73–5.31], respectively).

### Demand for leopard fur reduced due to synthetic furs

Sixteen of the 22 stakeholders we interviewed, including all 10 Lozi community representatives and six of seven conservation NGO staff, ranked Furs for Life Zambia as the first or second most influential driver of leopard recovery in the GKE. Interviewees agreed that the switch from authentic furs to synthetic furs reduced demand for leopard furs.

Community stakeholders stressed four attributes of synthetic fur that, in their opinion, accelerated adoption: they were readily available, distributed free of charge, visually appealing, and socially acceptable to the wider community (A1, A3–A5). Community stakeholders linked the difficulty in procuring authentic leopard furs to the rapid adoption of substitutes (A1, A3–A5). Interviewees stated that providing synthetic furs and conservation messaging fostered a cultural shift favoring leopard conservation (B4, B7, A9). This shift likely limited resistance from traditionalist sections of society and facilitated the wide adoption of synthetic furs (A4, A5, A6, A9, B2).

All community members and most conservation NGO interviewees (six of seven) opined that reduced market demand likely weakened the motivation for targeted poaching. A few interviews (A6, A8, A10) suggested that demand reduction impacted paddler‐driven targeted poaching and poaching by local poachers for sale to traders and paddlers. No interviewee suggested that synthetic fur availability had stimulated demand outside the Lozi community, countering a common substitution risk.

### Counter‐trafficking operations caused market disruption

Nineteen of the 22 interviewees representing all three stakeholder groups identified counter‐trafficking operations as a key causal factor for leopard recovery. Support was unanimous among conservation NGO stakeholders (seven of seven) and high among community (eight of 10) and DNPW stakeholders (four of five). Interviewees reported that the operations heightened the legal risks faced by offenders, disrupted trafficking networks, and removed prolific offenders through arrest and conviction (A5, A6, B3, B4, B6, C1–C3). By dismantling these networks, the interventions were thought to restrict poachers’ opportunities to market leopard furs, thereby lowering the financial incentive to kill leopards (B2, C2, C3).

Heightened probabilities of detection, arrest, and prosecution were also cited as major deterrents to poaching (B2, B6, C1–C3). Respondent B6 noted that visible court follow‐ups and publicized convictions further increased perceived risk among potential offenders. The incapacitation of key offenders through imprisonment was viewed as reducing ongoing poaching pressure on leopards and their prey (B4, B6, C3). Overall, interviewees perceived that elevated legal risks, disruption of trade networks, and the removal of key traffickers were critical to curbing illegal activity and supporting leopard population recovery.

### Counter‐poaching patrols reduced opportunity and increased perceived risks

Counter‐poaching patrols were considered a key driver of leopard recovery by 16 of 22 interviewees across all three stakeholder groups. Patrols were ranked the top intervention by most DNPW respondents (three of five) and conservation NGO staff (five of seven). Interviewees emphasized that wider patrol coverage and higher patrol frequency improved the detection of illegal activities, including leopard and prey poaching (B2, A8, B7, B5, C1–C4). Hiring local community scouts was viewed as pivotal, strengthening community support, information flow, and the timely reporting of offenses (B7, B4).

Greater detection raised the perceived likelihood of arrest and prosecution; together with the incapacitation of likely prolific offenders, this reportedly deterred would‐be poachers (A3, A9, B2, B3, B4, C1–C5). Patrol teams also directly disrupted poaching by seizing snares and firearms and regularly interrupting illegal activities (C1–C4). These actions increased the costs and effort required to poach, reducing its feasibility. Overall, stakeholders considered heightened detection, increased legal risk, and higher operational costs for offenders as central to the decline in leopard and prey poaching, facilitating leopard population recovery across the GKE.

Court‐monitoring data indicated that counter‐trafficking and counter‐poaching interventions led to a rapid and sustained increase in wildlife‐related cases filed each year, from 80 in 2018 to 213 in 2023 (an average annual rise of 21%), followed by a 35% decline in 2024 (139 cases). From 2018 to 2024, a total of 1069 cases were registered involving 1146 individuals, of whom 76% were convicted by the end of 2024.

### Integrated mechanism of change

**FIGURE 4:**
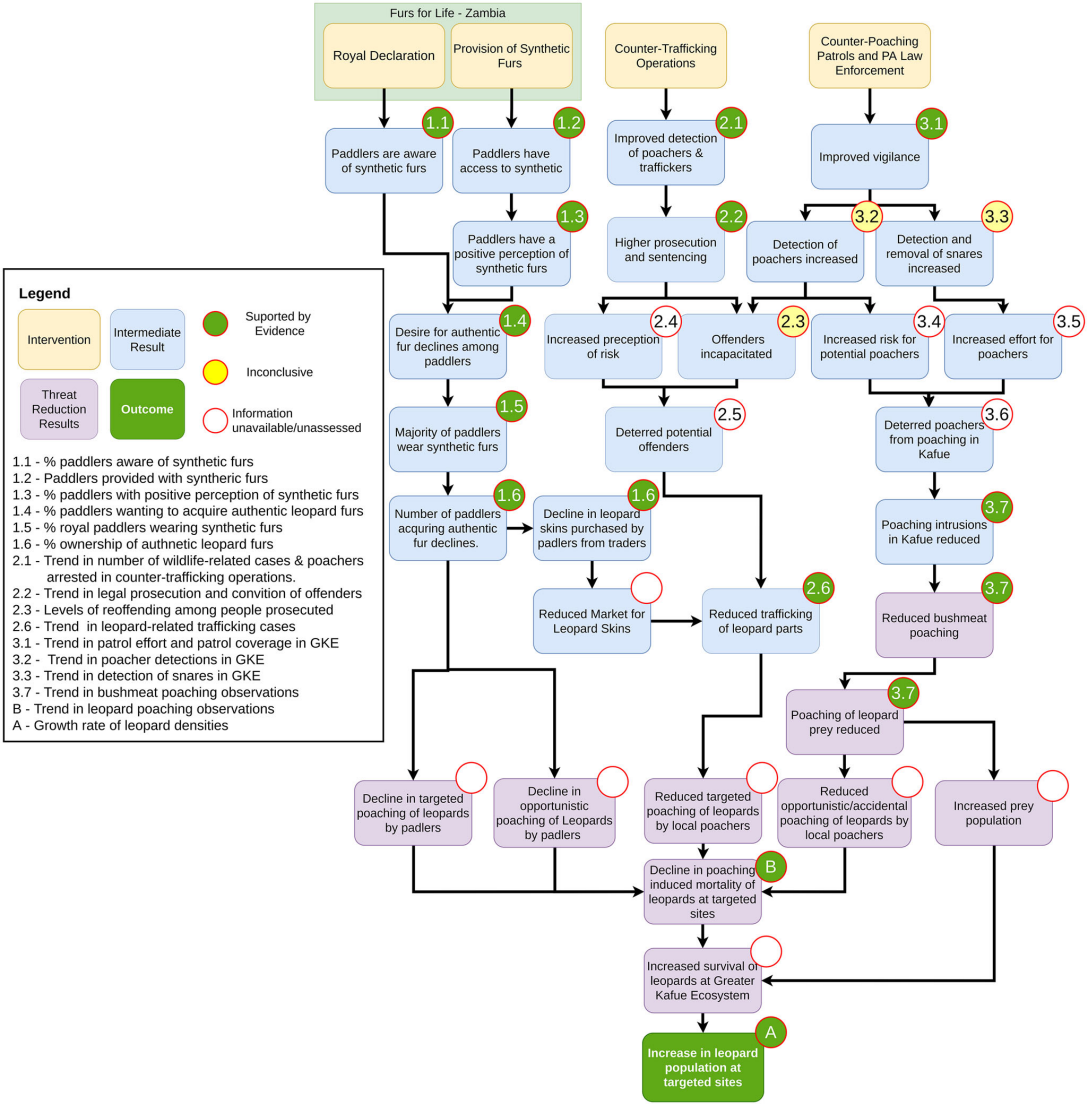
Integrated mechanism of change illustrating validated pathways for interventions leading to leopard recovery and evidence rating (GKE, Greater Kafue ecosystem).

A clear causal mechanism was evident: culturally endorsed substitutes eliminated ceremonial demand and reduced poaching incentives, and counter‐trafficking and counter‐poaching contributed to the recovery of leopard populations by disrupting trafficking and bushmeat poaching in GKE (Figure 4). A summary of evidence supporting the causal pathways is in Appendix S4.

Eck's four causality criteria indicated strong evidence of the effectiveness of the Furs for Life Zambia project (Table 1 ). The decline in leopard fur ownership, trafficking, and poaching occurred after the intervention began in late 2019, with the scale of the decline proportionate to the extent of synthetic fur adoption. Thus, the intervention meets three criteria fully and one partially, providing robust inferential support for its impact on leopard recovery.

**TABLE 1:** Eck's (2017) four criteria of causality applied to Furs for Life Zambia project.

Criterion	Evidence and conclusion
Decline in the problem comes after the intervention	Criterion fulfilled. Following the implementation of the Furs for Life Zambia project, a rapid and sustained decline in demand for authentic leopard furs was observed. Interview data revealed a 78% reduction in authentic fur ownership among paddlers from 2020 to 2024, with reported desire for authentic furs declining concurrently. Law enforcement data further corroborated these trends. Recorded poaching incidents across the Greater Kafue ecosystem (GKE) declined from a total of 14 incidents from 2018 to 2020, before the intervention, to two incidents recorded from 2021 to 2024. Court records similarly show a 75% decrease in leopard fur seizures during the same period (2018–2024), suggesting a marked disruption of the trafficking supply chain. Leopard population densities increased in all five monitored sectors from 2018 to 2024. Two sectors demonstrated a twofold increase in density, rising from approximately two to four individuals per 100 km^2^. The temporal alignment between the onset of the Furs for Life Zambia intervention and these demographic improvements, coupled with concurrent reductions in poaching and trade indicators, provides strong inferential support that the intervention contributed to demand reduction and population recovery outcomes for leopards in the GKE.
Level of intervention and the size of decline in the problem are related	Criterion fulfilled. Adopting synthetic furs led to a proportionate decline in leopard fur ownership. Decline in fur ownership, similarly, resulted in a proportionate decline in the trafficking of leopard furs.
Clear mechanism by which the intervention caused the decline	Criterion fulfilled. By distributing synthetic leopard furs through the Barotse Royal Establishment, Furs for Life Zambia intervention provided a culturally legitimate substitute for the primary ceremonial use of authentic leopard skins. The Litunga's decree and sustained outreach shifted social norms so that wearing synthetics became the accepted default, reducing paddlers’ willingness to buy, commission, or hunt for real skins. Reduced ceremonial demand likely lowered the expected rewards for targeted poaching. This demand‐side contraction was reinforced by concurrent counter‐trafficking and counter‐poaching that increased the effort, risk, and cost of supplying authentic skins, together reducing illegal offtake and enabling leopard population recovery across the GKE.
Alternative explanations rejected	Criterion partially fulfilled. Although intensified counter‐poaching patrols and antitrafficking operations likely contributed to reduced leopard mortality, the scale and timing of leopard recovery suggest that these efforts alone were insufficient to account for the observed outcomes. Leopard population recovery began in two of the five sectors before the adoption of SMART‐enabled patrols but shortly after the launch of the Furs for Life Zambia intervention in 2019, which included the royal directive mandating the use of synthetic furs. Moreover, the consistent and significant decline in leopard fur ownership and ceremonial use, over 70% from 2020 to 2024, coincided with widespread adoption of synthetic furs and was not preceded by similar trends in enforcement activity alone. These patterns align with the mechanics of demand reduction; however, supply‐side effects cannot be fully rejected.

**TABLE 2:** Overall leopard population growth (λ_overall_) for sites with density estimates derived from multisession spatial capture–recapture models.

Site	No. surveys	Mean survey area (km^2^)	Baseline density estimate (leopards/100 km^2^) (95% CI)	Baseline population size^a^	λ_overall_
Kafue National Park					
Kafue central	5	372	3.0 (2.1–4.3)	11.6	1.2
Kafue north	4	243	2.5 (1.5–4.1)	6.1	1.1
Kafue south	4	274	0.7 (0.4–1.3)	1.9	1.4
Game management areas					
Mumbwa	4	407	3.3 (2.3–4.7)	13.4	1.1
Mulobezi	4	262	0.9 (0.4–2.0)	2.4	1.4

^a^ Baseline population size was estimated by multiplying the estimated density (per square kilometer) from the first camera‐trap survey undertaken at each surveillance site by the mean survey area of that site.

### Moderators of effectiveness

Through the interviews, we identified two types of leopard fur users among prospective paddlers: early adopters and change‐resistant users. Early adopters composed 79% (218 of 276) of respondents expressing positive views toward the synthetic furs who rapidly adopted and used these instead of authentic furs. Quality and realism were shared as reasons for use by 52% of early adopters, with the conservation of leopards stated as important by 26%. Change‐resistant users, a smaller group comprising 14% (39 of 276) of respondents, expressed negative views toward synthetic furs, citing the need for preserving traditions and culture (85% of respondents).

Groups differed markedly in their stated desire to obtain authentic leopard furs: 67% of change‐resistant users compared to 16% of early adopters (*p* < 0.01). In 2020, the only year for which hunting data were available, 53% of change‐resistant users reported having hunted leopards. There was no evidence that level of education (*p* = 0.32) or employment status (*p* = 0.30) was associated with a particular user type. Demand and procurement of authentic furs by a small number of change‐resistant users prevented the project reaching 99% expected decline in authentic fur ownership by 2024.

### Economics of implementation

The total cost of implementing the leopard fur substitution intervention over 10 years (2020–2029) was estimated at USD691,678. This comprised an initial start‐up expenditure of USD313,558, incurred from 2018 to 2020 for infrastructure, community consultation, and design, production, distribution, and promotion of synthetic fur. Recurring maintenance costs, including staff salaries, community engagement, repairs, and monitoring, are projected at USD378,120 over the 10‐year implementation window.

Our analysis estimated that, in the absence of intervention, approximately 176 authentic leopard fur garments would be required each year to meet ceremonial demand among prospective paddlers. Based on the average yield of four garments per leopard, this translates to roughly 44 leopards killed annually. Over 10 years following the introduction of synthetic alternatives, and accounting for the rate of adoption and the longevity of synthetic garments, the intervention is estimated to prevent the use of 360 whole adult leopards (36 leopards/year), with a plausible range of 204 to 1310 leopards under conservative and optimistic scenarios, respectively. Compared with the total program cost, the cost per leopard use prevented was approximately USD1924 in the likely scenario of preventing the use of 360 leopards, USD528 in the optimistic scenario (1310 leopards), and USD3395 in the conservative scenario (204 leopards).

## DISCUSSION

Our evaluation indicated that the Furs for Life Zambia project reduced demand for authentic leopard furs and contributed to species recovery by offering culturally acceptable synthetic alternatives, supported by traditional leadership and additional enforcement efforts.

### Synthetic substitutes, reduced demand, and population recovery

Quantitative results from our paddler surveys revealed a 78% decline in authentic leopard‐fur ownership from 2020 to 2024. This sharp decline coincided with the provision of culturally endorsed synthetic furs, suggesting a targeted effect rather than a general market constraint. Reported trafficking of leopard furs fell by more than 74%, and average leopard density across the GKE increased by approximately 65%, indicating population‐level recovery. In most monitoring areas, density increases followed the rollout of SMART‐enabled patrols and the launch of the Furs for Life Zambia initiative. However, leopard recovery in Kafue South and Mulobezi GMA preceded SMART implementation, indicating that observed trends were not solely due to increased enforcement.

Leopard populations exposed to poaching and other human pressures show marked declines or persist at low densities (Gebretensae & Messele, 2022; Loveridge et al., 2022), and regional extinctions have been documented under sustained persecution (Rostro‐García et al., 2024). The contrasting trend observed in GKE, therefore, provides inferential support for the hypothesis that alleviation of human‐caused mortality led to leopard population recovery.

Stakeholder interviews supported the role synthetic furs play in demand reduction. Social legitimacy, visual similarity to authentic furs, free access, and endorsement by the Barotse Royal Establishment drove acceptance of synthetic furs. These findings are consistent with broader research showing that product substitutes are more likely to succeed when they fulfill functional, aesthetic, and symbolic needs (Rock & MacMillan, 2022; Thomas‐Walters et al., 2021). Similar dynamics have been observed with shark‐fin alternatives and lab‐grown rhino horn where perceived authenticity, social legitimacy and endorsement by trusted leadership were critical for uptake (Chen, 2017; Jeffreys, 2016; Zhou et al., 2021).

Interventions can produce displacement, where illegal activity shifts to other species, products, or locations, or diffusion of benefits, where preventive effects extend beyond the original target (Clarke & Weisburd, 1994; Cornish & Clarke, 2017). Here, rates of trafficking and use of other spotted carnivores did not change, suggesting intervention impacts were contained within leopard‐specific value chains.

### Requirements for complete adoption of synthetic substitutes

The initial rapid decline in authentic leopard fur ownership during 2020–2022, followed by a slower reduction thereafter, reflects a typical social diffusion trajectory in behavior change (Rogers et al., 2014). Socially responsive early adopters, catalyzed by the Litunga's endorsement, readily switched to synthetic furs. As substitutes gained visibility and social approval, adoption accelerated toward a new ceremonial norm among most paddlers. After 2022, the rate of decline slowed and fell below the zero‐procurement expectation, consistent with a late‐adoption plateau driven by change‐resistant users as demonstrated by our moderator analysis. Comparable behavioral segmentation is reported in wildlife product consumption studies, where individuals motivated by social image or practicality tend to change early, whereas those for whom use is linked to identity and cultural meaning are more resistant and require deeper normative reinforcement (Kidd et al., 2019; Thomas‐Walters et al., 2021). As 53% of change‐resistant users stated to have directly hunted leopards for furs, this group may have had different motives to early adopters. A successful leopard hunt resulting in four garments would open opportunities for sale to other paddlers, opportunities removed by the synthetic furs. Complete adoption of synthetic furs will require addressing the motivations and opportunities of change‐resistant users.

### Economics of product substitution with synthetic materials

The economics of implementing conservation interventions are rarely reported, despite this being critical to assess intervention cost‐effectiveness, proportionality, and suitability for illegal wildlife trade problems (White et al., 2022). Although vital for conservation‐dependent species, counter‐poaching and counter‐trafficking operations are extremely costly and less cost‐effective than alternatives. For instance, rhino dehorning in protected areas in South Africa accounted for 1.2% of $74 million spent on rangers, tracking dogs, cameras, better fences, and access control, but caused a 78% decline in rhino poaching where implemented, yielding a cost‐effectiveness of USD7133 per rhino saved (Kuiper et al., 2025). The synthetic furs project reached a likely cost effectiveness point of USD1924 per adult leopard use prevented over a 10‐year period for 360 leopards, suggesting that product substitution can be an economically viable strategy.

### Complementary supply‐side interventions and effectiveness of substitutes

The Litunga's decree and endorsement by the Barotse Royal Establishment transformed ceremonial norms, whereas rapid adoption of synthetic furs by paddlers conferred social legitimacy that initiated demand reduction and ecological recovery. This transformation, however, was not driven by substitution alone. It was reinforced by complementary supply‐side interventions and sustained outreach that together reshaped opportunity structures and social norms surrounding leopard use.

Intensified counter‐poaching patrols likely raised the perceived risk of detection among poachers, reducing access to hunting areas and opportunities to offend. Concurrent counter‐trafficking operations and prosecution efforts further increased the risk of arrest and may have disrupted trafficking networks by removing active intermediaries and key offenders—critical for influencing the change‐resistant fur users from continuing to hunt leopards. Empirical evidence indicates that deterrence is driven primarily by the certainty of apprehension and punishment, whereas severity has limited effect and swiftness remains less well substantiated (Nagin, 2013). In the GKE, increased detection and prosecutions, relatively high conviction rates, and publicity of cases likely enhanced the perceived certainty of sanctions, creating a deterrent environment further reinforced by collapsing consumer demand. Together, these actions increased the effort and risk required to offend and reduced expected rewards—mechanisms consistent with rational choice and situational crime prevention theory (Cornish & Clarke, 2017; Lemieux, 2014).

Stakeholders emphasized the role of sustained outreach—through community meetings, radio programs, and social media messaging—raising risk perception and normalizing the use of synthetic furs. This mirrors findings from behavioral research showing that interventions combining credible messaging, social reinforcement, and visible deterrents achieve greater compliance (Thomas‐Walters et al., 2021; Thomas‐Walters, McCallum, et al., 2023).

Collectively, these interlinked mechanisms illustrate a social–ecological transformation, where cultural adaptation, supply‐side controls, and ecological recovery acted as mutually reinforcing feedbacks (Olsson et al., 2004). The process also exemplifies Rumelt's (2011) notion of pivotal social leverage points—where focused action generates disproportionate system‐wide change—and thresholds, the tipping points beyond which transformation becomes self‐sustaining.

### Structured frameworks integrating qualitative insights

A key strength of this evaluation was the use of the EMMIE framework and the GEM to integrate quantitative and qualitative information. EMMIE enabled us to link observed effects to underlying mechanisms, implementation context, moderating factors, and economic costs. Ruling out alternative explanations and triangulation based on multiple lines of qualitative and quantitative evidence to derive a coherent theory of change enhances the validity of causal inference (Thomas‐Walters, Morkel, et al., 2023). This is especially important in demand reduction contexts, where changes in consumer behavior may also result from supply constraints, or consumer responses may be subject to bias.

Although qualitative methods have long been recommended for conservation evaluation (Salafsky et al., 2002), few studies rigorously apply them in interventions involving IPLCs. In such situations, where collective norms, deeply rooted beliefs, and traditional authority shape behavior, qualitative insights are crucial to understand how an intervention is received and endorsed by communities (Torrents‐Ticó et al., 2023). By capturing paddlers’ perspectives on tradition, social acceptance, and emerging norms, we gained deeper insight into both successes and resistance among more change‐resistant segments.

### Recommendations for future research

Multiple interventions overlapped spatially but varied in timing and intensity; this challenge is common in real‐world conservation settings where clearly demarcated pre–post or control–experimental designs are rarely feasible (Baylis et al., 2016). By triangulating behavioral, enforcement, and ecological evidence and applying GEM to assess competing explanations, this evaluation adheres to established principles of robust qualitative attribution (Zavaleta Cheek et al., 2023) and strengthens confidence in the observed linkages between substitution, reduced poaching, and leopard recovery.

This study benefited from the overt and socially sanctioned nature of wildlife product use, which minimized risks of concealment or misreporting. In contexts where such behaviors are covert or sensitive, increasing the potential for respondent bias or safety concerns, researchers may choose between the many Specialized Questioning Techniques (Cerri et al., 2021).

Embedding monitoring and evaluation systems that integrate suitable analytical frameworks, clearly defined metrics, and standardized protocols for data collection and management from the outset can greatly enhance evaluation and adaptation.

### Application to similar context

Ceremonial and ritual use of wildlife parts by diverse traditional societies spans continents and taxa (Alves et al., 2012; Williams et al., 2025). Despite cultural variation, many practices share core features: wildlife parts serve as potent cultural symbols, ceremonial use is visible and socially accepted, meaning resides in representation rather than material properties or commercial value, and traditional authorities retain the power to legitimize change.

Four key recommendations arise from this project's success that can guide teams tackling similar ceremonial wildlife‐use challenges and considering synthetics substitutes as an intervention: coownership of the intervention, codevelopment of substitutes, sustained awareness campaigns, and complementary supply‐side measures. Community leaders and traditional institutions must be involved in shaping the intervention strategy. Their endorsement lends legitimacy, ensures continuity, and reduces social risks tied to new substitutes—prompting faster adoption. When local institutions co‐own the process, the effort avoids perceptions of outside meddling in cherished traditions. Substitutes should be codeveloped in close partnership with traditional authorities and end users to craft alternatives that align with cultural, visual, and symbolic needs. Feedback from end users should be gathered, and refinements should be made iteratively. This co‐ownership and co‐development lay the foundation for localizing production, securing supply chains, and establishing sustainable financing—essential for long‐term success. Sustained, multichannel awareness campaigns should constitute in‐person engagements, radio broadcasts, and digital platforms to build and reinforce social acceptance. Consistent messaging normalizes synthetics, shifting norms over time. Supply‐side measures can be strengthened by implementing parallel compliance measures and removing or reducing illegal supply opportunities to support norm change, especially among resistant users. These actions complement awareness efforts and solidify adoption of synthetics. Our findings position culturally grounded product substitution with synthetic replicas as an effective, economically viable, and complementary addition to the conservation toolkit.

## Supporting information




**Appendix S1**: Paddler Questionnaire.

**Appendix S2**: Stakeholder Interview Details.

**Appendix S3**: Fermi Estimation Parameters for Cost‐per Leopard use prevented.

**Appendix S4**: Integrated Mechanism of Change Evidence Details.
